# Factors associated with latent tuberculosis among international migrants in Brazil: a cross-sectional study (2020)

**DOI:** 10.1186/s12879-021-06227-z

**Published:** 2021-06-01

**Authors:** Sonia Vivian de Jezus, Thiago Nascimento do Prado, Ricardo Alexandre Arcêncio, Keila Cristina Mascarello, Carolina Maia Martins Sales, Maysa Mabel Fauth, Nahari de Faria Marcos Terena, Raphael Florindo Amorim, Vania Maria Silva Araujo, Miguel Angel López Aragón, Ethel Leonor Noia Maciel

**Affiliations:** 1 Epidemiology Laboratory, Universidade Federal do Espírito Santo, Vitória, ES Brazil; 2 Graduate Studies Program in Public Health Nursing, Universidade de São Paulo, Escola de Enfermagem de Ribeirão Preto, Ribeirão Preto, São Paulo, Brazil; 3 Department of Health Sciences, Centro Universitário Norte do Espírito Santo, Universidade Federal do Espírito Santo, São Mateus, ES Brazil; 4 Department of Statistics, University of Rome La Sapienza, Rome, Italy; 5 Universidade Federal de Roraima, Undergraduate Course in Nursing, Boa Vista, RR Brazil; 6 Brazilian TB Research Network, REDE-TB, Rio de Janeiro, Brazil; 7 Pan-American Health Organization, Washington, USA

**Keywords:** Tuberculosis, International migrants, Society for the Reception of migrants, Latent tuberculosis, Refugee camps, Observational study

## Abstract

**Background:**

Migrants are a high priority group for TB control measures due to their high exposure to risk factors such as poverty and social vulnerability. The study aimed to identify factors associated with latent TB among international migrants living in four Brazilian state capitals. This was a cross-sectional study conducted in September and October 2020 in a sample of 903 international migrants living in four Brazilian state capitals: Boa Vista/RR (458), Manaus/AM (136), São Paulo/SP (257), and Curitiba/PR (52). Data were collected with a questionnaire consisting of open and closed questions on personal characteristics, information on TB, and use of preventive measures. Tuberculin skin test (TST) was performed, with reading after 72 h by trained nurses and using 5 mm induration as the positive cutoff. Chi-square test (*X*^2^) and Fisher’s exact test, both two-tailed, were used to compare statistically significant levels of association between the migrants´ sociodemographic characteristics, vulnerability, and latent TB infection (LTBI). Binary logistic regression was applied to calculate odds ratios and respective 95% confidence intervals. For all the tests, type I error of 5% was defined as statistically significant (*p* < 0.05).

**Results:**

Prevalence of LTBI among migrants was 46.1% in Manaus/AM, 33.3% in São Paulo/SP, 28.1% in Curitiba/PR, and 23.5% in Boa Vista/RR. Factors associated with latent infection were age, male gender, and brown or indigenous race.

**Conclusions:**

The study showed high prevalence of latent TB among international migrants.

## Introduction

With a focus for the first time on the global tuberculosis (TB) epidemic, heads of state and representatives met on September 26th, 2018, at the United Nations headquarters in New York and reaffirmed the commitment to end the global TB epidemic by 2030. Aligned with the Sustainable Development Goals (SDGs) through the Political Declaration of the UN General Assembly High-Level Meeting on the Fight Against Tuberculosis, they acknowledged the importance of appropriate prioritization of risk groups and other vulnerable persons [1].

The groups that received high priority include migrants, especially refugees, internally displaced persons, and persons living in situations of complex emergencies and poverty, acknowledging the higher TB prevalence in these groups [1].

Migration is a complex phenomenon, and the impact on migrants´ health generally depends on the form of transportation used, pathogenic or environmental exposures over the course of the transit routes, and epidemiological indicators in their home countries and places of destination [2].

International migration has reached high levels, with a 49% increase from 2000 to 2019, from 173 million to 272 million persons. This process has been accompanied by a change in the profile of migrants due to the new countries of destination, in addition to the larger proportion of women and children [3, 4], becoming a priority health issue in the Americas considering the unorganized and intensive migratory processes from Central America to North America (Mexico, United States, and Canada) and from Venezuela to the neighboring countries in South America and the Caribbean [4]. Specifically, in Brazil, some 100 thousand Venezuelans entered the country from January 2017 to November 2018 [5] across the border in the far North of Brazil.

The BRICS countries (Brazil, Russia, India, China, and South Africa) account for 40% of the global TB burden and mortality and for at least 50% of all cases of multidrug-resistant or rifampicin-resistant tuberculosis (MDR/RR-TB) [6]. However, there are still few studies in Brazil to assess the dynamics of TB transmission in border regions [7].

Migrants in situations of social vulnerability become particularly susceptible to TB. This situation relates directly to housing conditions, crowding, and type and place of work as some of the social determinants of the increase in TB cases and LTBI [7]. Prevention and diagnosis of TB or LTBI in this population becomes an important public health measure to reach the global TB targets, besides interrupting the transmission chain of the disease both in countries with low incidence and especially those with high incidence [7, 8].

Latent TB has been studied in international migrants [7], especially among refugees in Europe. However, in Latin America, including Brazil, few studies have focused on TB in migrants, despite the issue’s relevance for the policy to eliminate TB. A systematic review with meta-analysis found higher prevalence of LTBI among migrants in Europe than in Latin America, [9] perhaps because screening of LTBI in migrants in Latin America is still incipient. This calls for investigation, especially in the capital cities.

Based on the above, the study aimed to identify latent TB prevalence among migrants in four Brazilian state capitals and factors associated with latent infection, as well as to identify traits or prior history of contact with the disease.

## Methods

### Study design

This was a cross-sectional study with a survey design and direct data collection.

### Setting

The study was conducted in migrants living in four Brazilian state capitals, namely Boa Vista, state of Roraima (RR), Curitiba, state of Paraná (PR), Manaus, state of Amazonas (AM), and São Paulo, state of São Paulo (SP), which receive many migrants every year.

### Participants

The reference population is international migrants living in Brazil, independently of regular or irregular documentation status, and who agreed to participate in the study procedures. The exclusion criteria were individuals under 18 years of age and members of the same nuclear family of a person already selected, besides participants who had a history of active TB or even some presumptive symptom active TB, since these health conditions could be confounding factors in the investigation of LTBI. In case of more than one member of a family, we recruited the first one who had contact with our team.

### Procedures

Municipal governments, associations, and nongovernmental organizations that assist migrants and refugees were contacted. These institutions helped to publicize the project through a booklet called “Eye on TB” with availability of print and digital dissemination for social media and produced in the languages Portuguese, Spanish, and Warao (the indigenous ethnic group inhabiting northeastern Venezuela). This material provided information on TB, LTBI, the study procedures, and how to participate.

Those interested in participating contacted the project team by email, phone, social media, or lists posted at institutions that support migrants, according to guidelines provided in the booklet. This information was used to draw up a list of people potentially eligible for subsequent random selection. If more than one person from the same family was selected, a new participant was drawn. Then, contact was made by the research team, in which the migrants were invited to participate in a meeting by videoconference or video call via social media, to present the project. For those without access to video conferencing platforms or social networks that offer video calls, an explanation was made via telephone. If the migrant did not have a telephone, the contact was made in person, individually, with the presence of a researcher and a translator (if necessary), in an airy environment, with safe distance, using masks and providing alcohol gel. Recruitment of migrants began from August to October 2020, and a list was made of potentially eligible subjects for subsequent random selection. Each study participant was assigned an exclusive identification number consisting of a letter (the initial of the participating city) and four numbers.

### Data sources, measurement and variables

The study used two measurement instruments, an individual interview applying an instrument validated for study and not published, with the migrants´ demographic and clinical characteristics and health status and/or comorbidities. Participants were asked about their situation of vulnerability if they were unemployed, living in asylums, shelters, or hostels, self-identified as indigenous, incarcerated, or even homeless, besides their type of economic or work activity (when applicable), in accordance with the literature [10, 11].

They were also asked about current or past (lifetime) history of tuberculosis, and presence of TB symptoms at the time of the interview: presence of cough (yes or no), cough duration, coughing up blood or mucus, chest pain, or pain upon breathing or coughing, unintentional weight loss, fatigue, fever, or night sweats. Participants were classified according to the presence or absence of at least one of these symptoms. In case of positive symptoms, mainly cough more than 3 weeks, participants were referred to a medical specialist for examination.

Participants were inquired about their close contacts in relation to confirmed active TB diagnosis or presence of presumptive symptoms (index cases). TST was performed when the migrant agreed and the study criteria were met, with reading performed 48 to 96 h after application. For migrants that underwent TST, the test results were classified as ≥5 mm (reactive) versus < 5 mm (nonreactive), [12, 13] according to the Brazilian Ministry of Health guidelines and protocols. The 5 mm cutoff is based on higher sensitivity and higher odds of detecting *Mycobacterium tuberculosis* infection, as evidenced in previous studies and required for a screening test [13]. All participants were assessed and referred to the respective health services, according to Brazilian Ministry of Health guidelines [12].

Data collection used a structured interview with the questionnaire housed in the *Research Electronic Data Capture* (REDCap) system, which allowed direct data entry during the off-line interviews (after the internet connection, the questionnaires were transferred to the REDCap platform).

### Bias

Answers were collected through face-to-face interviews, conducted by experienced interviewers and in tandem with the TST. The questionnaire was pretested and validated by an experienced researcher and translated to Spanish when the migrants did not understand Portuguese. The interviewers were instructed to ask questions exactly as stated in the questionnaire and provide only non-directive guidance. A suitable and safe place was provided during the interviews for participants´ comfort and privacy. Data were collected through an online questionnaire hosted in Red-Cap system, and data quality was verified automatically by each interviewer and later electronically by the system. When data were not completed suitably, the interviewers were contacted to correct them.

### Study size

The study used probabilistic sampling. Sample size was calculated by estimating the prevalence of latent TB at 15 to 20%, based on estimates of the burden of latent TB infection in the Americas [14]. The other parameters were 90% power and 5% alpha error. We added 20% to correct for study design, plus another 20% for losses to follow-up [15]. Estimated sample size for the study was thus 880 subjects, of which 458 in Boa Vista, 35 in Curitiba, 151 in Manaus, and 236 in São Paulo, respecting the proportion of resident migrants in each of the capitals: 4.02% in Curitiba, 17.21% in Manaus, 51.97% in Boa Vista and 26.78% in São Paulo.

### Statistical methods

Descriptive statistics were performed to evidence differences regarding international migrants according to the cities under study. Additionally, Pearson’s chi-square test (χ2) or Fisher’s exact test was used considering the total cases, to test the association between LTBI (yes or no) and age, gender, occupation, and situation of social vulnerability (independent variables). Subsequently, variables with significant OR (*p* < 0.20) were included in the multivariate model through the backward stepwise method (likelihood ratio), obtaining the adjusted odds ratios (aOR). Variance inflation factor (VIF) was used to test for multicollinearity between the independent variables, therefore for evaluating whether the variance of a regression coefficient was inflated due to multicollinearity in the model. Since we used dummy variables with two or more categories, VIF less than 10 is acceptable [16].

Analysis of variance was applied to verify the best model, and Akaike information criterion (AIC) was also considered, in which the assumption of the best model is the one with the shortest distance in the probabilistic data generation process, which means reduction in the model’s variability and improving the model’s fit.

The models´ accuracy was analyzed using area below the receiver operating characteristic (ROC) curve and 95% CI. The ROC curve values were evaluated according to the literature [17]. Data were analyzed in RStudio, an interface of the R statistical software.

### Ethical aspects

The study was approved by the Institutional Review Board of the Federal University of Espírito Santo, the Brazilian National Commission for Research Ethics (CONEP) under case review no. 3.953.347, and the PAHO Ethics Review Committee (PAHOERC) under case review no. 0204.03. All subjects who agreed to participate signed a free and informed consent form in electronic format, with the guarantee of withdrawing from the study at any moment if they so desired.

## Results

Table 1 presents the selected study participants´ main characteristics. As shown in the table, most of the migrants were female, brown or black, and single, and the predominant age bracket was 25 to 59 years. Unemployment was reported by 55.5, and 70.0% were living in asylums, shelters, or hostels.

**Table 1:** Characteristic of the migrants recruited for the study, Brazil, 2020 (*n* = 903)

Variables	n	%
**Gender**		
Female	478	52.9%
Male	405	44.9%
**Race or color**		
Brown	416	46.1%
White	161	17.8%
Black	154	17.1%
Indigenous	80	8.9%
Others	31	3.4%
Creole	22	2.4%
Yellow	20	2.2%
Missing	19	2.1%
**Marital Status**		
Single	485	53.7%
Married/Stable Union	339	37.5%
Divorced	37	4.1%
Widower	11	1.2%
**Age Range**		
25 to 59 years	662	73.3%
18 to 24 years	182	20.2%
Over 60 years	52	5.8%
**Schooling**		
9 to 11 years	388	43.0%
5 to 8 years	228	25.2%
Over 11 years	168	18.6%
Up to 5 years	69	7.6%
**Occupation**		
Unemployed	501	55.5%
Informal worker	239	26.5%
Formal worker	79	8.7%
Never worked	14	1.6%
Undergraduate	11	1.2%
Worker	8	0.9%
Retired	7	0.8%
**Vulnerability**		
Asylum/shelter/hostel resident	632	70.0%
Not in a situation of vulnerability	211	23.4%
Indigenous	23	2.5%
Incarcerated	3	0.3%
Homeless	1	0.1%
Healthcare worker	1	0.1%
Missing	32	3.5%

Table 2 presents individual TB history, contact with an index case, and presence of cough. Prevalence of self-reported TB was 1.3% in Boa Vista, 1.9% in Curitiba, 1.5% in Manaus, and 6.2% in São Paulo. Among migrants who reported a history of TB, the majority had the disease more than 3 years before and in their home countries. BCG scar was observed in 84.9% of migrants in Boa Vista, 90.4% in Curitiba, 81.6% in Manaus, and 73.5% in São Paulo. Migrants were asked about the presence of cough (independent of time), and prevalence was 7.6% in Boa Vista, 5.8% in Curitiba, 6.6% in Manaus, and 14.4% in São Paulo. Mean duration of cough in days was 71.9 in Boa Vista, 14.9 in Curitiba, 7 in Manaus, and 80.7 in São Paulo.

**Table 2:** History of TB, contact with index case, and presence of cough among migrants in Brazil

	Boa Vista (***n*** = 458)	Curitiba (***n*** = 52)	Manaus (***n*** = 136)	São Paula (***n*** = 257)	Total (***n*** = 903)
**Variables**	**n (%)**	**n (%)**	**n (%)**	**n (%)**	**n (%)**
**Tuberculosis**					
Yes	6 (1.3%)	1 (1.9%)	2 (1.5%)	16 (6.2%)	25 (2.8%)
**How long ago**					
< 3 years	2 (0.4%)	0 (0.0%)	0 (0%)	7 (2.7%)	9 (1.0%)
≥ 3 years	4 (0.9%)	1 (1.9%)	2 (1.5%)	9 (3.5%)	16 (1.80%)
**Country where person had TB**					
Home country	5 (1.1%)	0 (0.0%)	1 (0.7%)	16 (6.2%)	22 (2.40%)
Brazil	1 (0.2%)	1 (1.9%)	1 (0.7%)	0 (0%)	3 (0.30%)
**BCG scar**					
Yes	389 (84.9%)	47 (90.4%)	111 (81.6%)	189 (73.5%)	736 (81.50%)
No	69 (15.1%)	5 (9.6%)	25 (18.4%)	67 (26.1%)	166 (18.40%)
Missing	0 (0.0%)	0 (0.0%)	0.00%	1 (0.4%)	1 (0.10%)
**Cough (regardless of duration)**					
Yes	35 (7.6%)	3 (5.8%)	9 (6.6%)	37 (14.4%)	84 (9.30%)
**Duration of cough in days**					
(Mean; SD)	71.9; 199	14.7; 0.6	7; 6.7	80.7; 212.3	67.9; 192.8
**Contact with TB case**					
Yes	62 (13.5%)	6 (11.5%)	28 (20.6%)	45 (17.5%)	141 (15.60%)
**TST test performed in migrant after contact**					
Yes	11 (2.4%)	1 (1.9%)	15 (11%)	26 (10.1%)	53 (5.90%)

Of the total, 2.8% had a history of TB and 9.3% reported cough, and a mean duration of cough was 67.9 days. All cases with cough for more than 3 weeks or another active TB’s presumptive symptom were refereed to health services for examination and were excluded for ILTB investigation.

Table 3 shows data on TST in the migrants. The test was performed in 98.5% of participants in Boa Vista, 61.5% in Curitiba, 94.1% in Manaus, and 59.1% in São Paulo. Since TST is an invasive test, the uptake varied between the four cities, as evidenced in Table 3. The rate of TST reactivity (≥ 5 mm) was also high, with 23.5% in Boa Vista, 28.1% in Curitiba, 46.1% in Manaus, and 33.3% in São Paulo.

**Table 3:** Number of TST performed and results in international migrants recruited in four Brazilian state capitals

Variables	Boa Vista (***n*** = 458)		Curitiba (***n*** = 52)		Manaus (***n*** = 136)		São Paulo (***n*** = 257)	
	n	%	n	%	N	%	n	%
**TST performed**								
Yes	451	98.5%	32	61.5%	128	94.1%	153	59.5%
No	7	1.5%	16	30.8%	7	5.1%	104	40.5%
Missing	0	0.0%	4	7.7%	1	0.7%	0	0.0%
**TST result (of those tested)**								
< 5 mm	345	76.5%	23	70%	69	53.9%	102	66.2%
≥ 5 mm	106	23.5%	9	28.1%	59	46.1%	51	33.3%

Although 764 migrants who had a TST, only 753 results were considered valid for analysis, mainly due to the migrant’s failure to return the test reading, besides some inconclusive results. Table 4 shows the results of the bivariate analysis, where the variables gender, color/ race, marital status, and social vulnerability were more significant and therefore were incorporated in the regression model according to the study criteria. Table 4 also allows identifying differences between migrants with negative versus positive TST.

**Table 4:** Bivariate analysis of factors associated with LTBI among international migrants, Brazil (2020)^a^

Variables	TST		***p***-value
	Negative n (%)	Positive n (%)	
**Gender**			
Female	297 (39%)	106 (14%)	0.10**
Male	232 (30%)	118 (15%)	
**Color/Race**			
Yellow	6 (1%)	5 (1%)	< 0.001**
White	111 (15%)	35 (5%)	
Creole	14 (2%)	3 (0.4%)	
Indigenous	26 (3%)	46 (6%)	
Brown	270 (35%)	85 (11%)	
Black	82 (11%)	42 (5%)	
Other	21 (3%)	8 (1%)	
Missing	4 (1%)	1 (0.1%)	
**Marital Status**			
Married/Stable Union	199 (26%)	86 (11%)	0.004**
Divorced	15 (2%)	18 (2%)	
Single	299 (39%)	119 (16%)	
Widow (er)	8 (1%)	0 (0%)	
**Age Range**			
18 to 24 years	113 (15%)	46 (6%)	0.96
25 to 59 years	389 (51%)	163 (21%)	
Over 60 years	31 (4%)	12 (2%)	
**Schooling**			
Up to 5 years	40 (5%)	24 (4%)	0.21
5 to 8 years	140 (18%)	61 (8%)	
9 to 11 years	231 (30%)	94 (12%)	
Over 11 years	104 (14%)	31 (4%)	
**Occupation**			
Retired	6 (1%)	1 (0.1%)	0.70
Unemployed	326 (43%)	134 (18%)	
Student	3 (0.4%)	4 (1%)	
Never worked	11 (1%)	3 (0.4%)	
Undergraduate	4 (1%)	1 (0.1%)	
Formal worker	41 (5%)	19 (2%)	
Informal worker	127 (17%)	59 (8%)	
**Situation of vulnerability**			
Indigenous	7 (1%)	11 (1%)	0.009**
Asylum/shelter/hostel resident	433 (57%)	168 (22%)	
Not in a situation of vulnerability	83 (11%)	37 (5%)	
Healthcare worker	0 (0%)	3 (0.4%)	
Missing	11 (1%)	6 (1%)	

^a^Applied Pearson’s 훘2 or Fisher’s exact test when the expected frequency was less than 5; **p < 0.2

Three models were constructed in the study according to the criteria. The first model considered the variables male gender, situation of vulnerability, single marital status, indigenous, brown, or black race, undergraduate student, and healthcare work as occupation. The second model considered male gender, indigenous or black race, undergraduate student, and healthcare work as occupation. The third model considered age, male gender, indigenous or black race, undergraduate student, and healthcare work as occupation (Table 5). According to the analysis, model 3 presented the best AIC and the lowest residual deviations.

**Table 5:** Analysis of variance (ANOVA) of the models tested in the study (2020)

ANOVA of Proposed Models				
	GL Residuals	Residual Deviations	Deviation	*P*-value
Model 1	748	854.78		
Model 2	750	855.59	−0.81	0.667
Model 3	749	850.72	4.86	0.027

Table 6 shows model 3, and the findings revealed that male gender increased the odds of LTBI by 43% and black color/race by 64%, while indigenous individuals showed five times higher odds of LTBI than non-indigenous individuals. Access to higher education reduced the odds of LTBI by 30%, and healthcare work was a risk factor for LTBI. The lowest VIF was 4.70 and the highest 7.75, for indigenous ethnicity and healthcare work as vulnerability, respectively. Based on the model selected for the study, the ROC curve was 67%, i.e., 72% sensitivity and 54% specificity, means satisfactory predictive value.

**Table 6:** Adjusted multivariate analysis of factors associated with latent TB infection among international migrants, Brazil (2020)

	Confidence Intervals				*p*-value
	Coefficient	aOR	2.50%	97.50%	
Intercept	−1.78	0.17	0.1	0.29	0.00
Age	0.013	1.01	1	1.03	0.03*
Male gender	0.355	1.43	1.03	1.98	0.03*
Vulnerability, Healthcare Worker	16.25	11.445.451.51	0	Inf.	0.97
Indigenous color/race	1.731	5.65	3.34	9.56	0.00*
Black color/race	0.495	1.64	1.07	2.51	0.02*
University education	−0.369	0.69	0.43	1.1	0.12

*p < 0.05

## Discussion

The study aimed to identify factors associated with LTBI among international migrants. The findings showed that age, male gender, and indigenous or back color/ race were associated with LTBI among these migrants, confirming the relationship between TB and vulnerability and inequities classically known and corroborated by the study. International migrants in situations of social vulnerability are a high-risk group for LTBI, due mainly to their exposure to vulnerabilities, with precarious housing conditions, crowding, often with poor health status and other diseases, and difficulties in accessing health services, frequently due to language and other barriers [18].

Although international migrants are a high-risk group for TB exposure, to the best of our knowledge, previous studies have not systematically quantified TB prevalence or examined risk factors for LTBI in this population in Brazil or in other developing countries with high TB burden. Using a multicenter cross-sectional design in four Brazilian state capitals, the study found a high prevalence of LTBI (TST ≥ 5 mm) among migrants in Brazil. Factors associated with LTBI were age, male gender, and brown or indigenous self-reported race/color, evidencing the association between latent TB and social determinants.

Although the results are important for global public health policies, the study has some limitations. First, it uses a cross-sectional design, so the temporal relationship between positive TST and *M. tuberculosis* infection could not be established. Second, there is no gold standard for detecting LTBI, so the estimated prevalence of LTBI may be affected by the test’s performance and the migrant’s own health status. Although these limitations are important, we contend that they are offset by the multicenter design, following the same operational standards to maintain the quality and standardization of the study’s data collection in the four sites.

The results show high prevalence of LTBI (TST ≥ 5 mm) in international migrants in Brazil, i.e., approximately 30% overall, or 23.5% in Boa Vista, 28.1% in Curitiba, 46.1% in Manaus, and 33.8% in São Paulo. A recent meta-analysis with studies published from January 2000 to August 2017 found 37% prevalence of LTBI among refugees and asylum-seekers, varying according to country of origin and continent of destination, and 28% among those who migrated to the Americas [9]. Although the search in the meta-analysis included the recent period of migratory crisis, no study was observed with migrants from the Americas, especially Latin America and specifically the Venezuelan population. In the current study, 77% of the migrants were from Venezuela, a country where the economic crisis has directly affected the health system since 2013, given the political crisis in that country in recent years [19].

A curious finding in the study was higher prevalence of reactive TST in Manaus than in the other three cities, possibly due to the number of indigenous Venezuelans (Warao ethnicity) who have taken refuge in villages in and around Manaus. The Warao are an indigenous people from northern Venezuela who have lived in the Orinoco Delta for centuries, in the state of Delta Amacuro and adjacent regions in the states of Bolívar and Sucre. They have traditionally become vegetable farmers, living in villages on stilts located in riverine areas and marine estuaries, as well as swamps and flooded forests around Manaus [20], poor areas lacking safe drinking water and basic sanitation, a relevant reservoir for TB as the literature has evidenced [21].

Since TB and latent TB infection are intrinsically related to living conditions, access to health services, and social determinants, prevalence of LTBI among migrants can be explained by the country of origin and their socioeconomic conditions upon arrival in Brazil. This is related first to the fact that the migrants come from countries where many infectious diseases are endemic and where they have often received limited healthcare before their departure [11, 22]. Most of the study’s participants are from countries where TB is endemic and have experienced situations of social vulnerability such as unemployment, as observed in the results. Second, most migrants participating in the study were living in shelters, another factor associated with LTBI. A study in a refugee shelter in southern.

Italy found 60% prevalence of LTBI among the 982 residents tested. The authors underscored that these migrants are a special group of immigrants, since specific risk factors such as overcrowding can expose them to higher risk of infectious diseases [23].The proposed multivariate model showed higher odds of LTBI in male, indigenous, and black migrants and those with low schooling. A study of refugees recently arrived in São Diego, California, aimed at determining the prevalence and treatment rates for latent TB infection, found a higher risk of LTBI in persons with low schooling [24].

It is not surprising that low schooling is associated with LTBI in refugees, since this variable has appeared in studies [25, 26] in the general population. The probable explanation is individuals´ low socioeconomic status. Most migrants in the current study are believed to have lived in places with intense social vulnerability and exposure to *M. tuberculosis*.

Host countries have the responsibility to offer migrants access to diagnostic methods and adequate treatment, besides meeting all their specific health needs in line with the targets of the Sustainable Development Goals (SDGs) to end TB. Urgent action is needed to guarantee access to early diagnosis and treatment among migrants, especially refugees. Most high-income countries have consolidated programs for screening and treating TB in migrants, including LTBI [26].

In Brazil, although the Unified Health System (SUS) guarantees access to public health services such as diagnosis and medication for treatment of latent tuberculosis, there is no targeted policy for TB control in migrant/refugee populations. Of the four cities participating in the study, Manaus in the state of Amazonas drafted the first plan in Brazil to care for Venezuelan migrants, including activities for TB.

Regarding limitations, it is important to highlight biases related to the study design, specifically to reverse causality, since it may be difficult to ascertain the temporal order regarding exposure and outcome, which were collected concomitantly. Another issue is exposure, since the study was unable to confirm precisely whether the migrants had been infected in their home countries (mainly Venezuela) or in Brazil, since both countries are equally affected by TB burden. Besides, study participants were residing in urban areas, and no approach or investigation was conducted in remote or rural areas, where TB burden may be higher and its control even more difficult. Another limitation was that TST not being performed in large proportions in two cities, possibly introducing a bias.

Migration is a highly dynamic and complex process, and migrants have moved around Brazil extensively, due mainly to the interiorization policy adopted in some cities, which poses challenge for their follow-up and TB prevention. The study evidenced high prevalence of LTBI among migrants. A high priority for health services and authorities should be to prevent these cases from developing active TB. All cases identified by the study as LTBI were referred to the health services in the respective cities. Future studies should include longitudinal follow-up of migrants to verify their health conditions, preventive treatment adherence, and progression to active TB.

Brazil is among the priority countries listed by the WHO [27] for expanding the global response to achieve universal access to TB diagnosis, treatment, and prevention. However, achievement of the TB control targets will require combined efforts on different fronts, including governments, civil society, human rights advocacy groups, and others, to accelerate access to prevention and care, as well as to monitor the actions´ progress [28] and include the specific problem of TB in migrants and refugees and implement specific initiatives for this population.

## Data Availability

The datasets used and/or analyzed during the current study are available from the corresponding author on reasonable request.
