# Correction to: Factors associated with latent tuberculosis among international migrants in Brazil: a cross-sectional study (2020)

**DOI:** 10.1186/s12879-021-06305-2

**Published:** 2021-06-21

**Authors:** Sonia Vivian de Jezus, Thiago Nascimento do Prado, Ricardo Alexandre Arcêncio, Keila Cristina Mascarello, Carolina Maia Martins Sales, Maysa Mabel Fauth, Nahari de Faria Marcos Terena, Raphael Florindo Amorim, Vania Maria Silva Araujo, Miguel Angel López Aragón, Ethel Leonor Noia Maciel

**Affiliations:** 1 Epidemiology Laboratory, Universidade Federal do Espírito Santo, Vitória, ES Brazil; 2 Graduate Studies Program in Public Health Nursing, Universidade de São Paulo, Escola de Enfermagem de Ribeirão Preto, Ribeirão Preto, São Paulo Brazil; 3 Department of Health Sciences, Centro Universitário Norte do Espírito Santo, Universidade Federal do Espírito Santo, São Mateus, ES Brazil; 4 Department of Statistics, University of Rome La Sapienza, Rome, Italy; 5 Universidade Federal de Roraima, Undergraduate Course in Nursing, Boa Vista, RR Brazil; 6 Brazilian TB Research Network, REDE-TB, Rio de Janeiro, Brazil; 7 Pan-American Health Organization, Washington, USA

**Correction to: BMC Infect Dis 21, 512 (2021)**

**https://doi.org/10.1186/s12879-021-06227-z**

Following publication of the original article [1], an error was identified in the text:

The text currently read:

Abstract

**Background:** Migrants are a high priority group for TB control measures due to their high exposure to risk factors such as poverty and social vulnerability. The study aimed to identify factors associated with latent TB among international migrants living in four Brazilian state capitals. This was a cross-sectional study conducted in September and October 2020 in a sample of 903 international migrants living in four Brazilian state capitals: Boa Vista/RR (458), Manaus/AM (136), São Paulo/SP (257), and Curitiba/PR (52). Data were collected with a questionnaire consisting of open and closed questions on personal characteristics, information on TB, and use of preventive measures. Tuberculin skin test (TST) was performed, with reading after 72 h by trained nurses and using 5 mm induration as the positive cutoff. Chi-square test (X2) and Fisher’s exact test, both two-tailed, were used to compare statistically significant levels of association between the migrants´ sociodemographic characteristics, vulnerability, and latent TB infection (LTBI). Binary logistic regression was applied to calculate odds ratios and respective 95% confidence intervals. For all the tests, type I error of 5% was defined as statistically significant (*p* < 0.05).

**Results:** Prevalence of LTBI among migrants was 46.1% in Manaus/AM, 33.3% in São Paulo/SP, 28.1% in Curitiba/PR, and 23.5% in Boa Vista/RR. Factors associated with latent infection were age, male gender, and brown or indigenous race.

**Conclusions:** The study showed high prevalence of latent TB among international migrants.

The text should read:

Abstract

**Background:** Migrants are a high priority group for TB control measures due to their high exposure to risk factors such as poverty and social vulnerability. The study aimed to identify factors associated with latent TB among international migrants living in four Brazilian state capitals.

**Methods:** This was a cross-sectional study conducted in September and October 2020 in a sample of 903 international migrants living in four Brazilian state capitals: Boa Vista/RR (458), Manaus/AM (136), São Paulo/SP (257), and Curitiba/PR (52). Data were collected with a questionnaire consisting of open and closed questions on personal characteristics, information on TB, and use of preventive measures. Tuberculin skin test (TST) was performed, with reading after 72 h by trained nurses and using 5 mm induration as the positive cutoff. Chi-square test (X2) and Fisher’s exact test, both two-tailed, were used to compare statistically significant levels of association between the migrants´ sociodemographic characteristics, vulnerability, and latent TB infection (LTBI). Binary logistic regression was applied to calculate odds ratios and respective 95% confidence intervals. For all the tests, type I error of 5% was defined as statistically significant (*p* < 0.05).

**Results:** Prevalence of LTBI among migrants was 46.1% in Manaus/AM, 33.3% in São Paulo/SP, 28.1% in Curitiba/PR, and 23.5% in Boa Vista/RR. Factors associated with latent infection were age, male gender, and brown or indigenous race.

**Conclusions:** The study showed high prevalence of latent TB among international migrants.

The original article has been corrected as well.

## References

[1] de Jezus SV (2021). Factors associated with latent tuberculosis among international migrants in Brazil: a cross-sectional study (2020). BMC Infect Dis.

